# Induction of neurotoxicity by organophosphate pesticide chlorpyrifos and modulating role of cow urine

**DOI:** 10.1186/s40064-016-3004-9

**Published:** 2016-08-12

**Authors:** Shelly Sharma, Pooja Chadha

**Affiliations:** Cytogenetics Laboratory, Department of Zoology, Guru Nanak Dev University, Amritsar, Punjab 143005 India

**Keywords:** Neurotoxicity, Brain, AChE, Cow urine, Chlorpyrifos

## Abstract

**Introduction:**

Organophosphate pesticides are among the most widely used synthetic chemicals for controlling a wide variety of pests and for domestic purposes. Among these chlorpyrifos (CPF) is the most extensively used pesticide throughout the world, including India.

**Objective:**

The present study was undertaken to examine the neurotoxicity induced by CPF and modulatory effect of cow urine as a natural antioxidant alternative to reduce the neurotoxic effects of CPF.

**Design:**

For this purpose LD_50_ was determined and one fourth of LD_50_ was selected (38 mg/kg body weight (b.wt)) for treatment of rats. The antioxidant level of cow urine was determined by ABTS assay.

**Results:**

Exposure to pesticides resulted in significant reduction in the acetylcholinestrase (AChE) activity (P ≤ 0.01). However, groups pretreated with cow urine had improved levels of AChE activity as compared to CPF treated groups.

**Conclusion:**

Thus, the present findings clearly show that oral CPF has the propensity to cause significant neurotoxicity in rat brains while cow urine treatment alleviates CPF induced toxicity to a greater extent. In addition, AChE can be used as a potential biomarker of toxicity associated with pesticide exposure.

## Background

The widespread use of pesticides in public health protection and agriculture pest control has caused severe environmental pollution and health hazards to target and non-target organisms (Noaishi et al. 2013). Their extensive application in modern agriculture requires an intensive investigation of the impact of these chemicals on the environment and public health. Due to the disposal of hundreds of millions of kilograms of pesticides each year, these agents must be analysed for their carcinogenic properties. The ever-increasing use of pesticides at an alarming rate and their unintended toxic effects on non**-**target organisms has raised an important global health concern. Organophosphate compounds are one of the most widely used insecticides accounting for approximately 50 % of the insecticides used globally. Though organophosphate pesticides have already superseded organochlorine pesticides owing to their rapid biodegradability and shorter persistence, their indiscriminate use affects the environment. CPF (*O*,*O*-diethyl-*O*-3,5,6 trichloro-2-pyridyl phosphorothioate) is a chlorinated organophosphate insecticide that has enjoyed widespread use in agricultural and domestic pest control. It is among the most commonly used broad-spectrum conventional pesticide to control a variety of pests in agricultural and domestic purposes. The toxic effects of CPF are attributed to the irreversible inhibition of the enzyme acetylcholinesterase (AChE) and pseudocholinesterase in target tissues that leads to the accumulation of acetylcholine in synaptic junctions thus preventing the smooth transmission of nerve impulse (Colovic et al. 2013).

Currently, naturally occurring antitoxic alternatives to minimise the toxic effects of these compounds are highly desirable. Cow urine or “gowmutra” has a unique place in Ayurveda Sangraha, the ancient scriptures. It has been described as the most effective secretion of animal origin and is known as “Sanjivini” (Dhama et al. 2005). Further, it’s considered as the best of all types of animal urine (including human) and possess various therapeutic uses (Pandey and Chunekar 2009; Singla and Kaur 2016). Due to its importance *cow urine* has been granted US Patent (No. 6896907 and 6410059.) for its medicinal properties, particularly as a bioenhancer and as an antibiotic, antifungal, and anticancer potential. Our body contains many micronutrients that give us strength for the activities of daily life. When we urinate, these micronutrients are flushed out of our body. Cow urine corrects the deficiency of these micronutrients in the body, maintains the balance of these substances, and even cures so-called incurable diseases (Pathak and Kumar 2003). It has innumerable medicinal properties and is considered valuable in treating various types of diseases. Due to its numerous restorative properties, traditional healers and practitioners of Ayurveda have used it along with other herbs for the treatment of various diseases such as diabetes, skin diseases, fever, epilepsy, anaemia, abdominal pain, constipation and arthritis (Nagda and Bhatt 2014). According to the studies of Kelly (1997) and Randhawa (2010), it is also known to generate bioenergy at a cellular level. Jerald et al. (2008) reported that cow urine has antioxidant and antimicrobial activities. Using in vitro models, antioxidant, DPPH radical scavenging and superoxide scavenging activity were tested, and cow urine’s impending use as a viable substitute for synthetic antioxidants was emphasized. Furthermore, cow urine has been found to possess antimicrobial and lipase activity, which could be the key factor for its usage as a medicine (Kumar et al. 2004). Additionally, it is rich in volatile fatty acids and antioxidants, which restrain the formation of reactive oxygen species responsible for DNA damage. Cow urine is able to cleanse the system of toxins and act as an anti-toxin that protects the body from various types of poisons (Jain et al. 2010). Sanganal et al. (2011) studied the efficiency of the wound-healing potential of cow urine by excision wound model. Though there are numerous claims of the efficiency of cow urine, few studies are available to support these claims. Similarly, its efficiency against pesticide-induced toxicity has been rarely explored. Consequently, in the present study, the potential of cow urine in modulating the neurotoxicity induced by CPF in the brains of rats has been investigated. Hence, the present study was designed to scientifically validate the neuroprotective potential of cow urine.

## Methods

### Chemicals

CPF (99 %) was purchased from Sigma Aldrich, St Louis, U.S. Cow urine was obtained from Divya Pharmacy,Haridwar, India, while all other chemicals used were of analytical grade and were obtained from Sisco Research Laboratory, Mumbai, India.

### Determination of antioxidant property of cow urine by ABTS radical scavenging activity

The ABTS radical cation decolorization assay was performed according to the method of Arnao et al. (2001) with some modifications. ABTS^+^ was generated by oxidation of ABTS with potassium persulfate. The stock solutions included 7 mM ABTS solution and 2.4 mM potassium persulphate solution. The working solution was then prepared by mixing the two stock solutions in equal quantities and allowing them to react for 14-16 h (overnight) at room temperature in the dark. The solution was then diluted by mixing 2 mL ABTS solution with 50 mL methanol to obtain an absorbance of 0.306 ± 0.01 units at 734 nm using a spectrophotometer. A fresh ABTS solution was prepared for each assay. 10 µL of cow urine sample was allowed to react with 2 mL of the ABTS solution, and the absorbance was taken at 734 nm after 5 min. The ABTS scavenging capacity of the compound was calculated as

$$\% \;{\text{Inhibition}} = \frac{{{\text{Absorbance}}\;{\text{of}}\;{\text{control}}\,-\,{\text{Absorbance}}\;{\text{of}}\;{\text{sample}}}}{{{\text{Absorbance}}\;{\text{of}}\;{\text{control}}}} \times 100$$

### Animals and care

Adult male albino Wistar rats, weighing 120 ± 20 g were used in the present experiment. All animals were housed in cages at room temperature (25 ± 2 °C) with a relative humidity of 50–60 % and on a 12-h light–dark cycle. The animals had free access to commercial pellet diet and water ad libitum. All experiments were performed according to the guidelines for the care and use of laboratory animals and were approved by the Committee for Purpose of Control and Supervision of Experiments on Animals reference number: 226/CPCSEA2 013/20). Animals were acclimated 15 day prior to the experiment.

## Experimental design

LD_50_ (152 mg/kg b.wt) was determined and one fourth of LD_50_ was selected for administration to rats. The animals were randomly divided into four groups: group 1(control): animals were administered corn oil only; group 2(Control): animals were administered with *cow urine* only; group 3(CPF treated): animals were administered with CPF (38 mg/kg of b.wt); group 4 (CPF + *cow urine* treated): animals were pretreated with 0.5 mL *cow urine* for consecutive 10 days and then orally administered with CPF (38 mg/kg of b.wt).

### Tissue preparation

The animals were sacrificed after 24, 48, and 72 h of treatment. After decapitation, brain was immediately removed; blotted, weighed, and washed using a chilled saline solution. One part of the brain tissue was minced, cut into small pieces, and then dried on filter paper and homogenized (10 % weight/volume) separately; in ice- cold 1.15 % KCl, 0.01 M sodium, potassium phosphate buffer (pH 7.4) in a Potter–Elvehjem-type homogenizer. Further, the homogenate was centrifuged at 18,000*g* for 30 min at 4 °C, and the resulting supernatant was used for the determination of AChE activity.

### Determination of acetylcholinesterase (AChE) activity in rat brain

AChE activity in rat brains was determined by using acetylcholine iodide as substrate according to the method of Ellman et al. (1961).

### Statistical analysis

The data were analysed using SPSS 11.0 for Windows. The significance of differences was calculated using one-way analysis of variance followed by Tukey’s procedure for multiple comparisons.

## Results

### ABTS activity of cow urine

The amount of ABTS reduced was quantified by measuring a decrease in absorbance at 517 nm. The cow urine sample significantly reduced the ABTS radicals. The sample showed significant antioxidant activities using 2,2′-azinobis-(3-ethylbenzothiazine-6-sulfonic acid) (ABTS). The cow urine sample was found to exhibit higher ABTS radical scavenging activity of 92.93 ± 0.32 %.

### AChE activity

**Fig. 1:**
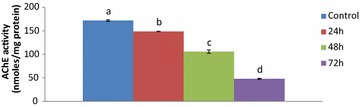
The effect of 38 mg/kg b.wt of CPF on AChE activity of rat brains at different time intervals. The results are shown as mean ± standard error. Means that do not share a *common letter* are significantly different (P ≤ 0.01)

**Fig. 2:**
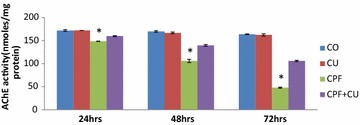
The effect of 38 mg CPF and CPF + CU on AChE activity of rat brains at different time intervals. Results are shown as mean ± SE. *Significantly lower as compared to CPF + CU treated group (P ≤ 0.01). CO: Corn oil, CU: Cow urine CPF: Chlorpyrifos, CPF + CU: Chlorpyrifos and cow urine

The results of the study are shown in the Figs. 1 and 2. A significant (P ≤ 0.01) decrease in AChE activity was observed at the end of the all-time intervals in the group treated with CPF only as compared with the control group. AChE activity continued to decrease with the passage of time. The activity decreased by 12.48, 37.67 and 71.76 % respectively, at 24, 48 and 72 h. However, statistically significant changes were observed in the *cow urine* + CPF- treated group. AChE activity increased significantly in the *cow urine* + CPF treated group as compared to the CPF-treated group at all time intervals. The values increased by 6.94, 24.19, and 54.70 % at 24, 48, and 72 h respectively (Figs. 1, 2).

## Discussion

Worldwide extensive use and poisoning by organophosphate pesticides, particularly in developing countries, including Indian sub-continent, is a serious health problem (Shetty et al. 2008). However, Jurewicz and Hanke (2008) emphasised the hazardous effects of organophosphate’s on the brain development of foetuses and children. AChE activity is considered a standard biomarker in toxicological studies of organophosphate pesticides. Organophosphates have been explicitly shown to exert detrimental effects on biological systems through the inhibition of AChE activity in the brain. The toxic effects of CPF are attributed to the irreversible inhibition of enzyme AChE which leads to a pathological excess of AChE and its decreased activity thus, preventing the smooth transmission of nerve impulse (Ricceri et al. 2006). In the present study, in vivo oral administration of CPF significantly reduced AChE activity in brain tissue within 24 h. Similarly, the short-term exposure of rats to CPF caused the significant inhibition of AChE activity in brains (Karasova et al. 2009; El-Demerdash 2011) and other tissues such as liver, kidney and spleen (Mansour and Mossa 2010). The reticence of AChE activity decreases cellular metabolism and nervous activity (El-Demerdash 2011). The degree of enzyme inhibition followed a positive correlation with various time intervals. Various studies suggest that both acute and chronic intoxication by CPF disturb the redox processes and lead to generation of ROS (Reactive Oxygen Species) which may further attack lipids, proteins, DNA, causing oxidation and membrane damage, enzyme inactivation and even cell death (Halliwell and Gutteridge 1999; Valko et al. 2004; Roszczenko et al. 2013). Organophosphate compounds undergo degradation in the body leading to formation of various transformation products. These transformed products are reported to be much more potent AChE inhibitors as compared to the thio OP’s (Colovic et al. 2013). So the observed time dependent decrease in the activity of AChE might be due to irreversible inhibition of enzyme AChE due to covalent binding of CPF/its degradation products to enzyme forming a stable enzyme- inhibitor complex.

However, the present study shows that the supplementation of cow urine along with the administration of CPF led to a significant increase in AChE activity, indicating that may have beneficial role in lowering the CPF-induced toxicity and may restore the AChE activity in CPF-treated rats. Earlier reports have shown that supplementation with cow urine attenuated the increased toxicity in rats intoxicated by carbon tetrachloride (Gururaja et al. 2009) and lindane (Nagda and Bhatt 2014). The results reveal that treatment with cow urine was effective in lowering the toxic effects of CPF. Thus, reduced levels of AChE indicate the effective neuroprotective efficacy of cow urine in the moderation of tissue damage. Matkovics et al. (1983) reported that AChE-inhibiting action of organophosphorus pesticides could be compensated by vitamin E. Furthermore, cow urine was found to be rich in various elements and compounds such as manganese, iron, silicon, calcium salts, minerals, lactose, chlorine, nitrogen, sulphur, phosphate, sodium, magnesium, citric, titric, succinic and carbolic acid, enzymes, creatinine, hormones, gold acids and vitamins A, B, C, D and E. The study by Krishnamurthi et al. (2004) showed that cow urine possesses antioxidant status of around 2.6 m mol, contributed mainly by volatile fatty acids (1500 mg/L) as revealed by the GC–MS studies. ABTS assay is an excellent tool for measuring the antioxidant capacity of hydrogen donating antioxidants. ABTS is a blue chromophore produced by the reaction between ABTS salt and potassium per-sulfate. As soon as cow urine was added to this pre-formed radical cation it reduced it to ABTS. In the literature, it has been observed that, ABTS reducing property is directly proportional to the amount of antioxidant activity. Therefore, the potent ABTS reduction observed in this assay might be due to the antioxidant compounds present in cow urine. Sachdev et al. (2012) revealed that cow urine contains volatile fatty acids such as acetic acid 2 propenyl ester, acetic acid methyl ester, 2,2,3,trichloro propionic acid, butanoic acid-3methyl, propyl ester, 1*H* indol-3-acetate, acetic acid phenyl ester and quinoline that may act as an antioxidants. This has been confirmed by the estimation of thiobarbituric acid, ascorbic acid, DPPH radical scavenging activity and ABTS assay. Furthermore, gold acids protect the body against oxidative stress that insults the nervous system (Jain et al. 2010). The presence of fatty acids and other antioxidants might have caused the observed protective effects.

The antioxidant potential might be contributing to the antitoxic effect, by preventing the formation of free radicals. Thus, the most probable reason for the neuroprotective potential of cow urine is its antioxidant properties and presence of various nutrients and minerals in its provision of beneficial effects. Cow urine may prevent the irreversible binding of CPF to AChE or helps in the regeneration of acetylcholine. From the present study it can be concluded that CPF has neurotoxic potential even at a low concentration and that cow urine has a significant anti-toxic effect.

## Conclusion

Hence, from this study, it was found that CPF is neurotoxic as revealed by decreased AChE levels and that cow urine has neuroprotective potential which might be due to its antioxidant activity. The highlight of the study is the competence of cow urine against the insecticide toxicity, which can open new insight in the therapeutic world for treatment of various disorders. The pharmaceutical industries can further explore its exquisite properties and its use for preparation of various types of drugs against various diseases. Further, cow urine may prove to be an effective co-remedy for the nervous system. Thus, sufficient regular consumption of cow urine by individuals, specially farmers, who are intermittently exposed to pesticides, is recommended as it can prove beneficial in inhibiting undesirable effects.
